# Echinoderms Metabolites: Structure, Functions and Biomedical Perspectives II

**DOI:** 10.3390/md20080492

**Published:** 2022-07-29

**Authors:** Vladimir I. Kalinin, Alexandra S. Silchenko

**Affiliations:** G.B. Elyakov Pacific Institute of Bioorganic Chemistry, Far Eastern Branch of the Russian Academy of Sciences, Pr. 100-letya Vladivostoka 159, 690022 Vladivostok, Russia

Echinoderms belong to the phylum Echinodermata (from the Ancient Greek words “echinos” (hedgehog) and “derma” (skin)). They possess radial symmetry, and have a unique water vascular (ambulacral) system. The phylum includes the classes Ophiuroidea (brittle stars), Asteroidea (starfish), Crinoidea (sea lilies or feather stars), Echinoidea (sea urchins) and Holothuroidea (sea cucumbers). All of them have a calcareous skeleton, which is reduced to ossicles in the sea cucumbers. Echinoderms inhabit all ocean depths and include more than 7000 living species. Echinoderms are a unique source of different metabolites that have a wide spectrum of biological activities. The representatives of Echinodermata have evolutionary acquired the exceptional mechanism of decreasing the level of free 5,6-unsaturated sterols in their cell membranes by sulfation of food sterols. Moreover, the starfish and holothurians are able to transform these 5,6-unsaturated sterols to stanols or 7,8-unsaturated sterols. This ability has arisen in parallel to the capacity of the representatives of these classes to synthesize and keep their own 5,6-sterol-dependent membranolytic toxins. Such toxins include triterpene oligoglycosides for the sea cucumbers and steroid oligoglycosides for the starfish. These metabolites have protective and ecological significance for the producers. Starfish and brittle stars biosynthesize numerous polyhydroxysteroids and their derivatives as food emulgators, which give them unique food plasticity. The echinoderms contain naphthoquinone pigments and carotenoids. The naphthoquinone derivatives are specifically characteristic of the sea urchins. The lipids of echinoderms are also uncommon, including cerebrosides and gangliosides characteristic of other Deuterostomia, namely, Chordata and Hemichordata. Echinoderms also contain lectins, glycan-specific glycoproteins that have immunity functions for the producers, glycosaminoglycans and fucoidans. Hence, plenty of unusual biologically active metabolites originated from the echinoderms [1].

This Special Issue begins from the articles dedicated to the sea cucumber triterpene glycosides. The first one by Silchenko et al. concerns the isolation, structural elucidation, cytotoxic activities and biogenesis of nine new non-holostane (having no lactone) triterpene glycosides from the Far Eastern sea cucumber *Thyonidium* (=*Duasmodactyla*) *kurilensis* (Levin), namely, kurilosides A_3_, D_1_, G, H, I, I_1_, J, K and K_1_. In addition to the native compounds, two desulfated derivatives, DS-kurilosides L and M, with interesting structural features were obtained from inseparable glycoside fraction. DS-kuriloside L has a trisaccharide branched chain, and DS-kuriloside M is characterized by a new hexa-*nor*-lanostane aglycone with a 7(8)-double bond instead of the 9(11)-double bond that is inherent for all other glycosidic aglycones from this sea cucumber. Their structures were elucidated by 2D NMR and HR-ESI-MS procedures. Five new carbohydrate chains and two new aglycones (having a 16*β*,20(S)-dihydroxy fragment and a 16*β*-acetoxy,20(S)-hydroxy fragment) were discovered in these glycosides. The analysis of the structural features of the aglycones and the carbohydrate chains of all the glycosides of *T. kurilensis* allows the creation of the schemes of their biosynthetic network. The cytotoxic activities of these compounds against mouse neuroblastoma Neuro-2a and normal epithelial JB-6 cells, as well as hemolysis against mouse erythrocytes, were investigated. The glycosides that have free hydroxyl groups in the aglycones were not active, whereas the other ones have moderate activities [2].

The second article by Silchenko et al. concerns chemical structures and cytotoxicity of triterpene glycosides from the Far Eastern sea cucumber *Psolus chitonoides.* The authors isolated four new triterpene disulfated glycosides: chitonoidosides E_1_, F, G and H. Two of them (chitonoidosides E_1_ and G) are hexaosides, differing from each other by the terminal (sixth) sugar residue, one is a pentaoside (chitonoidoside H) and one is a tetraoside (chitonoidoside F). The structures were elucidated using 2D NMR and HR-ESI-MS procedures. The most interesting structural feature of chitonoidoside G is the aglycone of a recently discovered new type, with an 18(20)-ether bond instead of a corresponding lactone. Rarely found terminal 3-*O*-methylxylose residue was a part of the carbohydrate chain of chitonoidoside E_1_. The sulfate group in the uncommon position 4 of terminal 3-*O*-methylglucose is a distinctive feature of chitonoidosides F and H. The hemolytic activities of all the studied glycosides, including previously isolated chitonoidoside E against human erythrocytes and cytotoxicity against several human cancer cell lines, such as adenocarcinoma HeLa, colorectal adenocarcinoma DLD-1 and monocytes THP-1, were also studied [3].

The third article by Zelepuga et al. concerning sea cucumber metabolites describes the structure–activity relationships (SARs) for a broad series of sea cucumber glycosides in relation to different tumor cell lines and erythrocytes. The results showed a very complicated character of the SARs for this class of compounds depending both on the structures of aglycones and carbohydrate chains and their combinations, including positions and number of sulfate groups. The most interesting part of the article is devoted to in silico modulation of the interaction of several glycosides from *Eupentacta fraudatrix* with model erythrocyte membranes by simulations of full-atom molecular dynamics (MD). The modeling revealed that the glycosides bound to the membrane through hydrophobic interactions and hydrogen bonds. The mode of such interactions may be very different and depends on the aglycone structure, especially the side chain structural peculiarities. Different mechanisms of glycoside/membrane interactions were found. The first one was realized through the pore formation (by cucumariosides A_1_ and A_8_), which was preceded by the bonding of the glycosides with the membrane cholesterol, sphingomyelin and phospholipids. The carbohydrate chain serves as an anchor bonding to the polar heads of phospholipids. Later-occurring noncovalent intermolecular interactions inside multimolecular membrane complexes and their stoichiometry differed for these glycosides because they were mainly dependent from the aglycone structures. Cucumarioside A_1_ had holostane aglycone with a 24(25)-unsaturated side chain penetrated to the outer membrane leaflet, which caused the formation of a “pore-like” assemblage. Cucumarioside A_8_ has hydroxy-groups at C-18 and C-20 instead of 18(20)-lactone, which promoted the initial stage of its integration to the membrane that was followed by the deepening of the aglycone to the outer membrane leaflet, causing the subsequent rearranging of the inner leaflet. Noticeably, powerful interactions between glycoside molecules inside the pore-like complex and phospholipids were observed for cucumarioside A_8_. The second mechanism was realized by cucumarioside A_2_ having a 24-OAc group through the formation of phospholipid and cholesterol clusters in the outer and inner membrane leaflets, correspondingly. The glycoside/phospholipid interactions were also more preferable than the glycoside/cholesterol interactions. However, the glycoside interaction agglomerated the cholesterol molecules from the inner membrane leaflet. The modulation of the interaction of cucumarioside A_7_, having the 24-hydroxyl group, with the model membrane revealed only weak bonding with phospholipid polar heads and the full absence of glycoside/cholesterol interactions. All the results correlated well with the experimental in vitro hemolytic activity of these substances. The obtained data open wide the possibility for understanding plenty of the experimental information concerning the membranolytic activities of the sea cucumber glycosides [4].

The next article by Kicha et al. concerns the isolation and structural elucidation of four new and one known steroid disulfates, including (20R)-7-oxo-24-methylcholesta-5,24(28)-diene-3β,21-di-*O*-sodium sulfate, (20R)-7-oxo-24-methyl-5α-cholest-24(28)-ene-3β,21-di-*O*-sodium sulfate, (20R)-24-methyl-7β-hydroxy-5α-cholest-24(28)-ene-3β,21-di-*O*-sodium sulfate, (20S)-cholesta-5,24-diene-3β,22-di-*O*-sodium sulfate, and (20R)-24-methylcholesta-5,24(28)-diene-3β,21-di-*O*-sodium sulfate from the starfish *Pteraster marsippus* [5]. The 2D NMR and HR-ESI-MS procedures were used for the structural elucidations. It is known that characteristics of the secondary metabolites of ophiuroids are mainly steroidal disulfated diols, triols, and rarely, tetraols that differ from other sulfated compounds of echinoderms in the positions of sulfoxy groups at 3α- and 21 in 5β-, Δ^5^-, and very rarely, 5α-cholestane cores. The common sulfated compounds from starfish have hydroxyl or sulfoxy groups occupying five or more positions [6]. It is of interest that similar ophiuroid-type polyxydroxysteroids, containing sulfoxy groups at 3α- (or 3β-) and 21 positions in 5α- or Δ^5^-cholestane nuclei were found in some species belonging to the family Pterasteridae (Velatida, Asteroidea), whereas the polyhydroxylated steroids and asterosaponins, common in other starfish, were absent in the starfish of this family. The currently obtained results correlate well with this trend. The cytotoxicities of a series of the isolated compounds were studied on models of 2D and 3D colonies of cancer cells, but the activities were weak or moderate [5].

The article by Malyarenko et al. is a review concerning the structure and biological activities of sphingolipids from starfish and sea cucumbers, namely, ceramides, cerebrosides and gangliosides. The gangliosides are the most complex sphingolipids characteristic of vertebrates, but have been found in the echinoderms also, which reflects a common origin of the Deuterostomia. The review comprehensively summarizes the data on sphingolipids of the sea cucumbers and starfish, which are the most studied among all the echinoderms, from the past twenty years. The structures, properties and peculiarities of biogenesis of ceramides, cerebrosides and gangliosides are discussed. The review makes obvious a great structural diversity and the presence of some unique structural features in these classes of starfish and sea cucumber lipids. It was shown that starfish and sea cucumber cerebrosides are perspective candidates for practical application in the human diet and in the composition of food supplements. Noticeably, the authors use in a non-traditional sense the term “molecular species” as an inseparable fraction of a certain class of lipids (really a sub-class) instead of the more common understanding of the term as a synonym for an individual substance characterized by a specific composition and the allocation of fatty acids in sphingolipids or the length of aliphatic chains in a sphingosine base. As a result, the phrases similar to, “Generally, about one hundred individual cerebrosides and their molecular species have been isolated from these animals”, are very characteristic of the review [7].

The article by Canadian researchers concerns characterization of unusual ether lipids and branched fatty acids from the viscera of commercially harvested North Atlantic sea cucumber *Cucumaria frondosa* and its seasonal variation [8]. The purpose of this investigation is to expand the nutraceutical use of the lipids from the viscera of this sea cucumber, but not the proteins, and different biologically active substances from the body walls and muscular bands only. The authors found that the highest total lipid content is in the winter season and diacylglycerols are predominant components. The triacylglycerols dominate in the summer season. The branched 12-methyltetradecanoic acid is a major component in diacylglycerols, and as a result, its total content seems to be maximal in the winter period also. The authors concluded that these observations should be taken into account during the harvesting of the sea cucumber. The authors used HPLC for the isolation of different classes of lipids and applied NMR spectroscopy for their characterization. They carried out the hydrolysis followed by derivatization of fatty acids and their analysis used traditional capillary GLC/MS. Such methodology seems to be quite adequate for the goals of the investigation.

The next review by Popov et al. concerns the application of MS-based metabolomics in the analysis of starfish and sea cucumber bioactive compounds [9]. It considers the use of metabolomic approaches for the study of polar steroids, triterpene glycosides and polar lipids. The authors note that elucidating the structure of starfish and sea cucumber metabolites is a very difficult task, comprising the isolation of individual compounds and followed by the application of modern MS and NMR techniques. They stressed that the use of an MS-based metabolomic method provides a possibility to study very complicated mixtures of echinoderm metabolites without the isolation of individual compounds. The authors describe the most characteristic features of MS-based approaches including sample preparation and MS analysis steps. Concluding the results of the MS-based metabolomic studies of secondary metabolites of starfish and sea cucumbers, the authors showed that the method makes it easy to carry out the detection and identification of polar steroids, triterpene glycosides and lipids in the organism-producer and to propose their preliminary structures. It allows the search for new biologically active molecules effectively and allows the authors to conclude their taxonomic distribution, biogenesis, and even biological functions. The MS-based metabolomic approach can be used successfully for comparing metabolomic profiles of different echinoderm species and populations for ecological, dietary, biosynthesis and chemotaxonomic studies [9]. It is necessary to note that the effectiveness of the discussed approach is highly dependent on the availability of the database containing the established structures of related compounds. Since the unambiguous identification and characterization of the compounds is a challenge for metabolomic research, it can be effectively applied only in combination with the isolation of individual substances and the elucidation of their structures.

The article by Ustyuzhanina et al. concerns the structural characteristics and blood anticoagulated activity of a series of fucose-rich sulfated polysaccharides from two Indo-West Pacific sea cucumbers *Bohadschia argus* and *Holothuria spinifera* collected from Vietnamese shallow waters [10], including fucosylated chondroitin sulfates FCS-BA and FCS-HS, along with fucan sulfates FS-BA-AT and FS-HS-AT. Extensive NMR analysis was used for the structural characterization of the polysaccharides. The authors showed that the fucosylated chondroitin sulfates contain a chondroitin core [→3)-β-D-GalNAc-(1→4)-β-D-GlcA-(1→]n bearing sulfated fucosyl branches at O-3 of every GlcA residue. These fucosyl residues were different in the pattern of sulfation. It was found that fucan sulfate FS-BA-AT is a regular linear polymer of 4-linked α-L-fucopyranose 3-sulfate. Because of the considerable sulfate content, FS-BA-AT is not active as an anticoagulant. The structure of fucan sulfate FS-HS-AT is a mixture of chains identical to FS-BA-AT and the other chains are built up of randomly sulfated alternating 4- and 3-linked residues of α-L-fucopyranose [10].

Thus, the articles published in the Special Issue largely reflect the structural diversity of echinoderm metabolites including triterpene glycosides and fucosylated chondroitin sulfates, as well as branched fatty acids, di- and triacylglycerols and other lipid classes from the sea cucumbers, polyhydroxysteroids from starfish and different classes of sphingolipids from sea cucumbers and starfish. Finally, the MS-based metabolomic approach, which is very helpful for the estimation of such diversity, is discussed. The materials from the Special Issue also illustrate the biomedical potential of the presented metabolites as cytotoxins and anticoagulants. The in silico approach broadens the possibilities to investigate the mechanisms of the action of membranolytic compounds.
